# The Complex Interplay of Malaria and EBV in Burkitt Lymphoma

**DOI:** 10.3390/cancers18132146

**Published:** 2026-07-03

**Authors:** Rosemary Rochford, Sam M. Mbulaiteye

**Affiliations:** 1 Department of Immunology and Microbiology, University of Colorado School of Medicine, Aurora, CO 80045, USA; 2 Division of Cancer Epidemiology and Genetics, National Cancer Institute, Bethesda, MD 20892, USA; mbulaits@mail.nih.gov

**Keywords:** Burkitt lymphoma, Epstein–Barr virus, malaria, *Plasmodium falciparum*

## Abstract

Burkitt lymphoma (BL) is an aggressive B-cell lymphoma endemic in children in regions of sub-Saharan Africa, where its incidence geographically overlaps holoendemic *Plasmodium falciparum* malaria and poorly controlled childhood Epstein–Barr virus (EBV) infection. Despite decades of research, the precise mechanistic synergy between these two pathogens remains incompletely defined. This review synthesizes current epidemiological, immunological, and molecular evidence to propose an integrated model for the etiology of endemic BL. We outline a paradoxical, dual-edged relationship wherein EBV infection during infancy may provide a short-term child survival advantage against severe malaria while simultaneously increasing the long-term oncogenic risk in B-cells infected by EBV. *P. falciparum* infection triggers polyclonal B-cell activation, increasing the probability of an activation-induced cytidine deaminase (AID)-mediated *c-MYC* translocation in proportion to the recurrent parasite burden. Concurrently, EBV expands within this B-cell pool and modulates the host immune response, potentially through viral interleukin-10 (vIL-10), to prevent lethal malarial inflammation. At the cellular level, EBV provides a critical “second hit” when it establishes latency I infection that rescues *c-MYC*-translocated B-cells from apoptosis. This framework explains why BL manifests as a “tumor of malaria survivors,” peaking in incidence years after the highest-risk period for malaria mortality. Ultimately, this model underscores that malaria control is a critical form of cancer control and highlights key future directions for validating these pathways in prospective clinical studies.

## Simple Summary

This review describes current research on a cancer called Burkitt lymphoma. It is a cancer that occurs mostly in children living in sub-Saharan Africa, where *Plasmodium falciparum*, the parasite that causes malaria, can be transmitted year-round, and children are infected with Epstein–Barr virus (EBV) at a very early age. The focus is on research evaluating the causes of this cancer and, in particular, how these two pathogens, EBV and *Plasmodium falciparum* malaria, work together to increase the risk for this cancer. We propose a model whereby BL is more likely to occur in children who have survived multiple episodes of malaria, and EBV provides the second hit by rescuing B cells that have the *c-MYC* oncogene translocation. If this model is correct, controlling malaria in children will also reduce the burden of Burkitt lymphoma.

## 1. Introduction

In 1958, Denis Burkitt described a rapidly growing pediatric malignancy of the jaw and abdomen that would come to bear his name: Burkitt lymphoma (BL) [1]. Now understood to be a mature germinal center B-cell lymphoma, BL was first defined by its unique epidemiology: an “endemic” form in children across sub-Saharan Africa (SSA) and Papua New Guinea, and a “sporadic” form that was also observed in adults in the rest of the world [2]. While extensive genetic sequencing has since revealed that all forms of BL are molecularly defined by a characteristic *c-MYC* translocation, the epidemiological puzzle of the endemic form has persisted. Endemic BL’s geographical distribution is strongly correlated with regions of intense *Plasmodium falciparum* malaria transmission, and nearly all cases (over 95%) are positive for Epstein–Barr virus (EBV) [3].

For decades, EBV and *P. falciparum* have been recognized as the two most critical cofactors in the etiology of endemic BL. However, the central question has always been how these two pathogens, one asymptomatic and the other lethal, synergize to drive malignant transformation. Early research established the ecological overlap [4,5,6], but recent mechanistic studies have begun to shed light on precisely how *P. falciparum* infection disrupts the EBV life cycle and creates a cellular environment ripe for oncogenesis [7,8,9,10,11,12]. This has shifted the paradigm from simply describing an association to understanding the intricate biological pathways that enable children to survive the lethal effects of malaria but succumb to the oncogenic effects of EBV.

In this review, we will focus on the recent research that unravels the complex interplay between the parasite, the virus, and the cancer. By synthesizing epidemiological observations with molecular data, we will build a model that explains how the host’s interaction with malaria and EBV leads to the development of endemic BL in children in SSA.

## 2. Burkitt Lymphoma Epidemiology

### 2.1. Geographical Distribution and Environmental Links to P. falciparum

The geographical pattern of BL in SSA closely aligns with the endemicity of *P. falciparum* [2], which is the most consistent epidemiological evidence for a link between BL and malaria. The highest incidence rates are concentrated in the so-called “lymphoma belt” in the equatorial region of SSA, which spans from countries in West Africa across to countries including Kenya, Malawi, Tanzania, Uganda, Cameroon, and portions of Mozambique on the eastern coast of southern Africa [2,13]. The high BL incidence areas are characterized by their suitability for sustained high transmission of *P. falciparum*, with BL concentrated in those areas where transmission lasts for more than six months of the year and tapering off in areas with shorter seasonal transmission [2,5].

Within this lymphoma belt, BL incidence is high in low-lying areas and low in high-altitude areas or areas with a dry arid climate where malaria transmission is low or seasonal. These relief and climatic features explain the substantial variability of BL rates across nearby countries. For example, BL rates in low-lying Malawi are 11-times higher than in Kenya and 48 times higher than in Ethiopia, which sit at high elevations (>5000 ft above sea level). Similarly, BL rates in Chad and Mali, which are arid and have seasonal malaria for less than 6 months in the year, are 11 to 14-times lower than in Malawi [13]. While this evidence points to a strong correlation between sustained malaria exposure and BL, cases are also documented in regions with low *P. falciparum* prevalence [14], suggesting that not all cases are directly linked to heavy malaria.

### 2.2. Non-Malarial Co-Factors of BL

Although *P. falciparum* and EBV are the best-established environmental cofactors of BL, cases manifesting in “mini clusters” that are independent of malarial or EBV patterns have prompted hypotheses that there are additional cofactors influencing BL risk in malaria and EBV-exposed children. For example, case clusters reported in the Bwamba districts of Uganda in the 1960s [15] and in Malawi in the 1990s [16] coincided with epidemic activity of arboviruses like Chikungunya and Onyong-nyong, suggesting that these viral infections may influence BL activity in populations primed by malaria. BL cases have also been linked to spatial patterns of plants, such as *Euphorbia tirucalli*, whose sap can induce EBV reactivation and chromosomal abnormalities in EBV-infected B cells [17]. The association of these co-factors with BL and elucidation of the precise mechanisms by which they contribute to BL, including via B-cell proliferation and enhancing EBV viral replication, is a promising area of future research [16].

### 2.3. EBV Positivity in BL Cases in SSA

In SSA, EBV plays a central role in BL etiology. EBV DNA is detected in most childhood BL cases [3,18,19], with tumor cells harboring an average of 30–50 clonal copies of the virus [20]. The clonality of EBV in tumor cells suggests that EBV infection preceded, and probably contributed to, the final malignant event. The proportion of EBV-positive tumors varies from near universal (~95%) in East African cases to about 60% in other case series, such as in Rwanda and Nigeria, where up to 40% of cases are EBV-negative [21,22]. These EBV-negative cases represent a rare form of BL that molecularly resembles the “so-called sporadic” BL, which is more commonly found outside of Africa [23]. Recent molecular studies have revealed that EBV is a defining factor in the molecular makeup of BL [2,24], creating distinct subtypes that are consistent globally, regardless of where cancer appears.

### 2.4. Age-Related Risk Patterns

The risk of developing BL with age is biologically interesting. Cases are rare in children under the age of three, with risk rising quickly to peak between the ages of 6 and 9 and then decreasing thereafter [2,25]. Although this age pattern is thought to apply only for cases seen in SSA and is attributed to childhood exposure to *P. falciparum* and EBV, similar age patterns are observed in pediatric BL in the US, where malaria is absent, and EBV infection occurs during adolescence [26]. For example, analysis of childhood BL cases aged 0–14 years in the United States Surveillance, Epidemiology and End Results (SEER) during 1992–2005 showed a mean age at diagnosis was 7.8 years (standard deviation [SD] 3.7). Most cases (27%) were diagnosed in children aged 3–5 years, followed by children aged 6–8 years (25%), and only 6% occurred in children aged 0–2 years. This age pattern was like the cases recorded between 1973 and 1977 in the US, although the inclusion of adults skews the mean age [27]. The similar age-patterns of pediatric endemic and sporadic BL suggest a fundamental factor common to both settings, while local co-factors influence population incidence. One possible common factor might be age-related changes in B-cell mass [28]. Bone marrow studies of healthy subjects show that the percentage of CD20-positive B-cells (from which BL originates) peaks in the first decade, then decreases thereafter, which correlates with the age-specific BL incidence peaks and subsequent age-related decrease in BL risk both in endemic and sporadic settings [28].

While BL is predominantly viewed as a pediatric condition, adult BL cases occur in SSA [13]. The frequency of EBV in adult BL cases in SSA is less certain. One study reported a slightly lower prevalence of EBV in BL among adults (~78% positive) compared to the near-universal presence seen in pediatric cases [29]. A confounding variable in understanding the etiology of adult BL is the association with human immunodeficiency virus (HIV) infection [30,31] and the challenges in identifying HIV+ BL in SSA cancer registries [32]. However, analysis of aggregated data from multiple SSA countries indicates that cases occur during the fourth and fifth decades of life, a pattern that more closely resembles the age distribution of BL observed outside of Africa.

Adult BL may be under-ascertained in SSA because of a misperception of BL as a pediatric condition, with adult cases being diagnosed as non-specified large B-cell lymphoma. Diagnosis of adult BL in SSA may require a clinical and diagnostic paradigm shift to suspect and correctly diagnose these cases so that they are given correct treatment for BL that is different from that used in other aggressive lymphomas to achieve optimal outcomes.

The under-ascertainment of adult BL also means the disease’s true incidence is likely underestimated, leading to skewed cancer registry data. Accurately identifying older patients in SSA is necessary to understand the full scope of BL and to develop appropriate clinical protocols for all ages. These adult cases also open questions about whether they are malaria-driven, whether immunity to malaria is responsible for the lower BL risk in adulthood, and whether the second peak is related to waning of immunity to malaria, EBV, or other factors. Furthermore, questions arise about whether prevention strategies centered on malaria control in children may be sufficient to address the burden of BL in the adult population.

### 2.5. Sex-Related Disparities in Incidence

BL predominates in males (up to four times higher) [2], but with variation in the reported male-to-female ratio by the anatomic site of the tumor and age. The ratio is higher for tumors involving the face or head and lower for those located in the abdomen, and higher in children below 5 years, and decreases in older children [2]. The biological basis for male predominance remains unexplained. Given that sex hormone levels and exposure to *P. falciparum* and EBV are similar in pre-pubescent boys and girls, these factors, per se, may not explain the observed sex differences. However, more studies are pointing to differences in response to infection with the parasite by sex. For example, a recent study of controlled human infection found that males were more likely to have a delay from the time of infection to the detection of malaria parasites in the blood [33] suggests that a sex-related response to *P. falciparum* occurs. A caveat relative to understanding risk for BL in children is that this study was conducted in adults in whom hormonal and exposure characteristics may vary by sex. More intriguingly, a longitudinal cohort study in a malaria-endemic region of Uganda followed both children and adults to detect the onset of new *P. falciparum* infections and resolution of infections using quantitative PCR and amplicon deep sequencing of parasites in the blood to track infections [34]. They observed that females (both children and adults) cleared parasites faster in the blood relative to males. Perhaps the extended length of infection in males compared to females contributes to the increased risk for BL in males.

## 3. Malarial Epidemiology in the Context of BL

**Figure 1:**
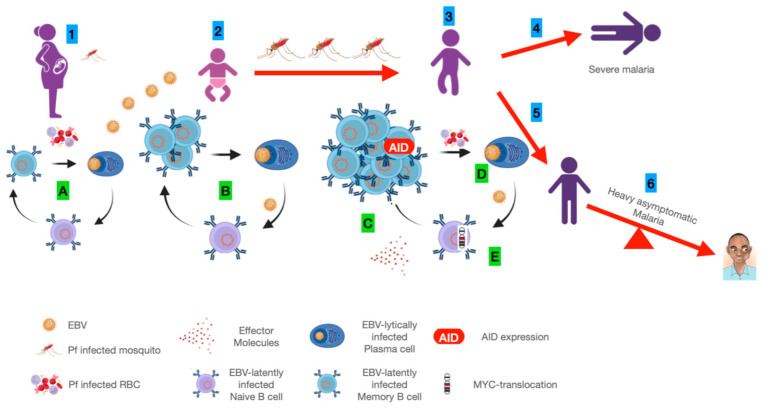
Model for the interplay between *P. falciparum (Pf)*, Epstein-Barr virus (EBV), germinal center B cells, and the development of Burkitt lymphoma (BL). The top cartoons depict different ages considered from in-utero (1), infancy (2), early (3) and later childhood (4–6) with progressive exposure with *P. falciparum* malaria throughout childhood. (4) A child suffers from severe malaria and risk of mortality or (5) develops immunity to severe disease and subsequent chronic asymptomatic parasitemia leading to increased risk for BL (6). The lower cartoons (A–E) depict malaria-EBV interactions in the germinal center B cells at different ages, with progressively increasing degrees of B-cell expansion, EBV lytic replication, EBV load, hierarchical development of memory B cells immune response to malaria. (A) EBV life cycle in B cells goes from latently infected naïve B-cell to latently infected memory (m)B cell to reactivation from latency in a plasma cell. Pf malaria upregulates EBV lytic replication in pregnant women, thereby increasing the chance that infected mothers will shed EBV in breast milk and infect their babies shortly after birth. (B) EBV life cycle in infants affected by Pf malaria and results in higher number of EBV- infected mBC. (C) B-cell proliferation increases the available targets for EBV infection and replication, raising the viral load. Pf induces AID via effector molecules (BAFF, *Pf* DNA/TLR9 ligation) in B cells increasing risk for c-MYC translocation. EBV helps modulate the child’s immune response to malaria, preventing lethal excessive inflammation and improving overall survival. (E) Sustained asymptomatic malaria infection maintains pressure on germinal center B-cells, increasing the probability that EBV will infect a germinal B cell that has acquired *MYC* rearrangement, constituting a second hit that enables the abnormal B-cell to evade apoptosis triggered by negative feedback loops and progress to BL (See details in text).

To get at the role of *P. falciparum* in BL etiology, it is first necessary to provide a brief background on *P. falciparum* epidemiology. *P. falciparum* transmission in different geographic areas ranges from sporadic (unstable, less intense) transmission to seasonal transmission during certain months, or to stable transmission characterized by recurrent exposure and repeated malaria infections throughout the year (also referred to as holoendemic malaria) [35,36]. Regardless of the transmission intensity, a child’s innate and adaptive immunological response to their infection determines their fate (death or survival) [37]. A child whose immune system is unable to control the *P. falciparum* parasites has a high risk of developing high parasitemia and severe, acute complications of malaria (e.g., cerebral malaria, severe malarial anemia) and a high risk of death as a result (Figure 1) [38,39,40]. Children less than 5 years of age are at the highest risk of morbidity and mortality associated with *P. falciparum* infections, particularly in holoendemic malaria regions [36]. Such children do not live long enough to accumulate the necessary oncogenic events for BL to develop. *P. falciparum* elicits protective immunity from severe disease after a few infections [40,41], but individuals remain susceptible to milder episodes and anemia as well as asymptomatic parasitemia during years of continuous exposure, during which time recurring infections and illness occur [42,43,44]. Interestingly, older children were found to be infected similarly to children under 5 years, but their parasite load was more likely to be lower, associated with genetically complex infection, to be asymptomatic (without fever), and to remain detectable for a longer period [45,46,47]. The early acquisition of adaptive clinical and parasite immunity in children living in malaria hyperendemic communities enables them to control and survive multiple infections in their high transmission intensity environment [41] but also sets them on the path toward potential BL development (Figure 1). Most *P. falciparum* infections are asymptomatic with low or moderate parasite load detectable by standard microscopy or rapid diagnostic tests or at levels that are sub-microscopic, i.e., parasite load below 100 parasites/mL [48,49]. The peak incidence of BL of about 7 years of age [2] coincides with the age when children have a lower risk of malaria but remain at risk of asymptomatic complex infections [47,50].

## 4. Malaria and Host Immune Function

As noted above, inability to control *P. falciparum* infection leads to uncontrolled parasite proliferation with acute symptoms, including severe anemia, cerebral malaria, malaria prostration, which become mild as natural immunity is acquired [51]. Anemia, due to the lysis of infected red blood cells (RBC) as well as other mechanisms, is a common consequence of *P. falciparum* infection [52]. Lysis of RBCs releases substantial amounts of heme into circulation [53]. Although low-level infection is often asymptomatic because of the absence of fever [54], it comprises the majority of infections and can result in mild or moderate anemia [55,56] and can exacerbate undernutrition, increase susceptibility to other infections, and cause mortality when chronic [57].

Because children who do not survive malaria die, those who recover develop a strong immune response that enables them to survive potentially hundreds of *P. falciparum* infections throughout their childhood [58]. Survival of each subsequent infection is necessary for BL risk to be realized (Figure 1). Each *P. falciparum* infection triggers an incremental, polyclonal B-cell activation to fight the infecting parasite clone. Repeated infections, enabled when parasites switch to expressed multicopy genes (e.g., *var*, *rif*, *stevor*) for immune evasion, keep the child’s germinal centers in a state of near-constant, high-level B-cell proliferation, altered B-cell homeostasis, and B-cell activation. This leads to elevated levels of B-cell activating factor (BAFF, also known as Blys) and hypergammaglobulinemia [59]. This large expansion of the B-cell pool, constantly cycling through germinal centers, dramatically increases the statistical probability of an activation-induced cytidine deaminase (AID)-mediated abnormality involving the c-MYC translocation [2]. More cell divisions mean more opportunities for this primary oncogenic event to occur. Interestingly, *P. falciparum* DNA is a TLR9 ligand that can bind to TLR9 on B cells [60], providing critical secondary signals that block the apoptosis (cell death) pathways to a B-cell that has already acquired the c-MYC translocation found in BL [61,62,63]. As will be discussed later in this review, these direct and indirect effects of *P. falciparum* on the immune system may interact with EBV in germinal center B cells to contribute to the emergence of EBV+BL.

## 5. From Ecological Association Between Malaria and BL to Mechanistic Insights

Although the geographic overlap of high BL incidence with holoendemic *P. falciparum* malaria [6,64,65] was confirmed in micro-geographical studies [14,58,66], the biological link has been harder to demonstrate in individual-level studies. Evidence from case–control studies [67,68] demonstrate elevated antibody titers to *P. falciparum* schizont extract in children with BL compared to age-matched hospital-based controls, but the use of schizont extract limits the ability to identify specific *P. falciparum* proteins associated with risk. To address this issue, studies have measured antibodies to diverse *P. falciparum* antigens, e.g., *P. falciparum* erythrocyte membrane protein 1 (*Pf*EMP1) or SE36, showing that the association may be more nuanced. For example, elevated antibodies to SE36, which is a blood-stage malaria vaccine target, were associated with decreased BL risk [69]. Conversely, elevated antibodies against HRP-2, which are a surrogate of *P. falciparum* burden, were associated with elevated BL risk [69]. In another study evaluating 14 *Pf*EMP1 antigens [70], BL cases had a lower breadth and intensity of IgG reactivity, suggesting that children exposed to heavy malaria may calibrate their immune response to achieve control infection to tolerable levels without the excessive inflammatory response that drives pathology of severe symptoms [70,71,72].

Because antibody data are insufficient to quantify the number of previous *P. falciparum* infections, one recent study modeled high-resolution spatial data of the number of *P. falciparum* annual infections per child within a population and population risk of BL [58] across six regions in Uganda, Tanzania, and Kenya. BL risk was associated with the estimated life-time *P. falciparum* infection, but the risk became significant after a high number of infections (over 50), increasing by 39% per 100 additional infections [58]. These results highlight the large number of repeated infections before BL onset and suggest that a reduction in the infection burden may decrease BL risk.

This possibility was suggested by a study conducted in the Mara region of Tanzania in the 1980s to use chloroquine doses to suppress malaria in children aged 5–10 years [73]. Survey results demonstrated a dramatic (~75%) decrease in the incidence of BL in children after two years of the program, but the declines were not sustained when the program ended. In the early 2000s, international efforts were launched to control malaria in Africa through the widespread distribution of insecticide-treated bed nets (ITN), the establishment of indoor residual spraying for mosquitoes, and the introduction of rapid diagnostic tests and artemisinin-based combination therapy [74]. These efforts significantly decreased malaria transmission intensity throughout much of Africa [75] such that by 2020, areas previously considered holoendemic were now experiencing much reduced burden of malaria. Anecdotal reports that the incidence of BL has also decreased have been confirmed in observational studies showing a direct correlation between reducing malaria burden in SSA and declining incidence of BL. For example, the overall BL incidence declined by about 9.6% between 2010 and 2016 in a study conducted in Uganda, Tanzania, and Kenya, coinciding with long-term declines in *P. falciparum* burden in these countries [58]. Another study in Kilifi, a coastal region of Kenya with holoendemic *P. falciparum* transmission and high BL incidence, showed a concomitant decrease in hospital-based BL cases and clinical malaria diagnosed at the local hospital [76]. Over a 3-decade period (1990–1999, 2000–2009, 2010–2020), they saw a significant decline in both parasite prevalence and parasite density of any child (1–14-year-old) admitted to the hospital. This decline correlated positively with a decline in BL incidence rates.

On a larger scale, Schmit et al. [77] performed a meta-analysis based on published studies of BL incidence rates in sub-Saharan Africa between 1990 and 2023 and compared rates before and after the introduction of ITN. BL rates were 44% lower in the period after ITN compared with before, and after adjusting for potential confounders. A 1% increase in mean ITN use in the past 10 years before BL was associated with a significant 2% reduction in BL incidence rates. Together, these studies suggest that BL is not a complication of just a few discrete acute *P. falciparum* infections but a complication in children who have suffered and recovered from many malaria infections during childhood. This model raises a central question: why do children who recover from recurrent *P. falciparum* infections succumb to BL?

## 6. Malaria Modulates EBV Persistence Across Childhood

One reason for the high risk of BL is EBV, a DNA virus that infects most of the population worldwide. The virus persists as a latent infection in B cells for the lifetime of the host. EBV is periodically reactivated during lytic infection, leading to shedding in saliva, where it is transmitted to new hosts [78], shed in breast milk in mothers, where it can be transmitted to babies [79], and is shed in blood, where it infects new B cells to increase viral load. In SSA, EBV infection in children is asymptomatic, whereas in Western countries, infection delayed until young adulthood can result in infectious mononucleosis, a severe complication associated with fever, extreme fatigue, and swollen lymph nodes, but is self-limiting after 2–4 weeks [80]. In SSA, many children are infected with EBV by 1 year of age [81,82].

Because *P falciparum* infection has profound effects on germinal center B cells, the question becomes how does *P falciparum* affect the EBV life cycle in B cells? Could this be a key risk factor tying *P. falciparum* and EBV to increased risk for BL? Comparative studies in SSA on the impact of *P falciparum* on EBV persistence found that children in a malaria-endemic region were infected with EBV earlier in life than children from a region with unstable transmission, resulting in higher and variable viral loads over time [81,83]. The variability in viral loads was linked to EBV reactivation, with each malaria episode resulting in increasing levels of antibodies to the EBV VCA and EAd lytic proteins [84].

There is consistent evidence that children with acute malaria experience a sharp increase in EBV load, up to 5000 genome copies/10^6^ PBMC [10], but the number of EBV-carrying B cells in the acute malaria samples decreases to basal levels in the recovery samples [12]. EBV DNA is readily detected in the plasma of children with acute malaria [85,86,87] but is detected in relatively fewer children following recovery from acute malaria [85,87]. These results suggest that EBV reactivation during malaria is transient, particularly in children with uncomplicated malaria [88].

In a recent study in Kenya, EBV measured in plasma and saliva was assessed for methylation profiles to help distinguish the source of viral DNA, i.e., virion, which is lytic viral DNA and is unmethylated, versus cell-associated EBV DNA, which reflects cells that are latently infected and is methylated. The results showed that the effects of malaria on EBV were age-dependent and different in the systemic (plasma) or mucosal (saliva) compartments [87]. Consistent with the results of Jayasooriya et al. [89], the EBV load was not statistically different in peripheral blood cells from children with clinical malaria compared to community controls. However, when age was considered, there was a negative correlation between the age of a child and EBV load in peripheral blood in children with clinical malaria. Similar results were reported in the studies of Njie et al. [10] and indicate that in younger children with clinical malaria, there is a more pronounced effect and expansion of EBV-infected cells.

Combined, the results suggest that continued *P. falciparum* infection upregulates B-cell proliferation, which provides a fertile ground for EBV to infect, replicate, and establish persistent high EBV viral loads in early childhood (under age 5). The repeated *P. falciparum* infections would then result in the observed increased EBV load [11]. Thus, the lower age at EBV infection and significantly higher viral burden observed in African children compared to what is seen in Western populations may be attributed to the absence of malaria, leading to low-level stable latent infection (<5 EBV+ B cells/10^6^ B cells in peripheral circulation) [90,91]. However, while the burden of *P. falciparum*/EBV burden is highest in children below 5 years, the observed BL incidence is lowest in children under 5 years of age [87]. BL risk peaks in children between the ages of 6 and 8 years who have better control of *P*. *falciparum*, have low risk for severe malaria, and apparently have lower EBV viral load [92,93,94].

## 7. *P. falciparum* and EBV Lytic Reactivation

Given the higher frequency of EBV-infected B cells in children with heavy exposure to malaria and evidence of persistent viremia, what is the underlying mechanism linking *P. falciparum* and EBV? Several clues come from an expanded knowledge of the effects of *P. falciparum* on B cells, which can increase EBV viremia through both direct and indirect mechanisms. Lysis of *P. falciparum-infected* cells leads to high levels of heme during malaria [53,95]. B-cells can internalize heme as well as synthesize their own [96]. Heme binds to the B-cell transcription factor, Bach-2, resulting in its degradation [96]. Bach-2 transcriptionally represses Blimp-1, a transcription factor that promotes B-cell differentiation to plasma cells. Thus, loss of Bach-2 allows Blimp-1 transcription and subsequent plasma cell differentiation [97], which causes lytic reactivation of EBV+ B cells [98] and increased viral shedding of EBV into circulation. Using the Mutu I BL cell line, Burnet et al. [99] found that treatment with hemin (the oxidized form of heme) resulted in degradation of BACH2, plasma cell differentiation, and reactivation of EBV from latency. In addition, *P. falciparum* may directly increase EBV viremia by directly stimulating EBV reactivation or expansion of EBV+ B cells during malaria infections. *P. falciparum* was also shown to induce EBV reactivation through binding of its CIDR1α domain of the *P. falciparum* membrane protein 1 to CD36 on Mutu I EBV+ B-cell line [100,101]. The increased EBV viral load and polyclonal activation of the EBV-positive B-cell pool and enhanced survival set the stage for increased risk of EBV+ BL. Together, these studies point to a mechanism for how infection with *P. falciparum* can increase EBV load by inducing reactivation and consequently re-infection and expansion of a new pool of EBV+ B cells, increasing EBV load. Another mechanism for EBV upregulation may be via immunosuppression of EBV control. Children living in malaria-endemic regions have suppressed EBV-specific CD4 and CD8 T cell responses (reviewed in [102]). In addition, a recent study [103] found that the effect of *P. falciparum* during acute malaria on both CD4 and CD8 resulted in a shift from a Type 1 IFNγ response to a regulatory T cell- based IL-10 response, suggesting a possible mechanism for loss of immune control of EBV-infected cells and consequent increases in viral load.

## 8. *P. falciparum* and Generation of BL Cell Precursors

The chromosomal translocation of MYC onto the heavy or light chain immunoglobulin loci results in high unregulated levels of MYC expression, an early key molecular lesion in the development of BL [2]. An emerging consensus is that the *MYC* translocation is caused by the enzyme AID. AID is an essential enzyme in B-cell antibody maturation, causing both somatic hypermutation and immunoglobulin class-switching to increase antibody diversification and affinity [104]. Several lines of evidence suggest that malaria increases the expression of AID in B cells, potentially increasing the chance for *MYC* rearrangement and the number of mutations within and outside *MYC*. For example, repeated infection of mice with the murine *P. chaubadi* resulted in B-cell lymphomas [105]. These lymphomas had the c-MYC translocation and phenotype consistent with BL. Furthermore, tumorigenesis induced by repeated *P. chabaudi* infection was dependent on AID. A second study using a transgenic AID reporter mouse model showed that *P. chaubadi* could induce aberrant AID activity in both germinal center and non- germinal center B cells [106].

However, overexpression of MYC in normal B-cells triggers feedback loops that initiate apoptosis [107]. How then do B cells that have already acquired a c-MYC translocation evade apoptosis? One explanation is that *P. falciparum* increases levels of the cytokine B-cell activating factor (BAFF, also known as Blys) [108,109], thereby increasing signaling of the BAFF-receptors (R) that are expressed exclusively on B cells [110]. BAFF increases B-cell survival through decreasing expression of pro-apoptotic Bak and increasing anti-apoptotic Bcl-2 and Bcl-x [111]. While this mechanism could explain apoptosis evasion of *MYC*+ B-cells and enable progression to malignancy, the relationship has not been shown in vivo.

Another possible explanation is that EBV infection delivers the critical “second hit” by expressing proteins that block apoptosis. Studies by Paschos and colleagues [112], found that the EBV protein EBNA3C can epigenetically modify the host genome and methylate the promoter for BIM, an anti-apoptotic protein. Moreover, a high level of methylation of the Bim promoter region in BL biopsies was observed, suggesting that EBV drove epigenetic repression. The role of EBV is also supported by whole genome sequencing (WGS) data from over 230 BL tumors, where EBV+ BL but not EBV- showed evidence of high levels of AID expression and a higher tumor mutational burden in EBV+ BL from both malaria and non-malaria endemic settings, suggesting that EBV upregulates AID, which is critical in malignant transformation [113].

Both *P. falciparum* and EBV have been shown in separate studies to induce expression of AID in B cells [8,9,114,115]. AID is elevated in the peripheral blood of children living in malaria-endemic regions with a high risk of BL [116]. Ariera et al. found expression of AID protein in CD19+ B cells in children with acute malaria, and levels of AID were sustained following resolution of malaria infection [9]. Moreover, they demonstrated that AID was translocated to the nucleus of the cell, indicating that the AID protein was functional. Torgbor et al. [8] found a higher frequency of AID+ B cells in the germinal centers in tonsils of children from a malaria endemic region of Ghana, and at higher levels compared to tonsils in US children. It was not determined if the AID+ cells in African children were also EBV+.

In the study by Torgbor et al. [8], B cells were incubated with an extract of *P. falciparum*-infected RBC and evaluated AID expression. *P. falciparum* extract from RBC was able to induce AID but was insufficient to induce high levels of AID unless cells were also stimulated with anti-CD40L and IL-4. They concluded that hemozoin induced AID via TLR9 ligation. An important caveat to that conclusion is that unless hemozoin is treated with DNase, it is likely that extracts of hemozoin contain *P. falciparum* DNA, which has been shown to be a TLR9 ligand [60]. In addition to the direct effects of parasite products on B cells, treatment of CD19+ B cells with BAFF, CpG ODN (to stimulate TLR9 ligation), and EBV resulted in a synergistic elevation of AID protein expression [9]. The role of EBV is also supported by DNA methylation studies of BL, showing frequent DNA hypermethylation of recurrently mutated genes in EBV+ BL (e.g., *CCND3*, *GNA13*, *TP53*, and *USP7*) [117].

These results have provided a unique insight into how EBV, acting as a second hit, might compensate for the lower mutation frequency of driver genes in EBV+ BL by providing alternative “don’t die” signals to cells that have acquired c-MYC translocation as a primary hit. Based on this model, *P. falciparum* infection triggers a massive, polyclonal B-cell activation to fight the parasite. Repeated infections keep the child’s immune system in a state of near-constant, high-level B-cell proliferation, dramatically increasing the statistical probability of an AID-mediated c-MYC translocation as a function of the mean number of cell divisions. The vast number of B-cells increases the targets of EBV infection, replication, and the chance of EBV infecting a B-cell that has already acquired the c-MYC translocation [118].

## 9. Model for *P. falciparum* and EBV in the Etiology of BL

Despite the plausible mechanistic link between *P. falciparum*, EBV, and *MYC* in the development of BL, the paradox is that BL occurs in immunologically competent survivors of *P. falciparum*, the deadliest infection of children in SSA, remains [119]. We present a model that combines both the observations from epidemiology studies with mechanistic cell models (Figure 1, Table 1). In populations where malaria is endemic, a child’s immunological response to their initial infections determines their fate. This response includes antibodies acquired prenatally from their mother, which likely shape their response in the first 6 months of life. In addition, since EBV infection is established early and expands exponentially with each *P. falciparum* attack, the model should account for why few BL cases are observed in children below 5 years when both oncogenic exposures are at their peak.

We propose a multi-step pathway for how *P. falciparum* contributes to BL development (Figure 1; Table 1). In step 1, children with strong innate and/or adaptive immune response experience recurrent *P. falciparum* infection, which induces polyclonal B-cell activation primarily to fight immune-evasive parasites and prevents death from acute complications. The intensity of repeated infections proportionately influences the intensity of B-cell proliferation and the statistical probability of developing an AIDS-mediated c-*MYC* translocation. Coincidentally, the expanded B-cell population increases targets for EBV infection, EBV replicating cells, and establishes a high EBV viral load. In mothers with malaria, this can lead to shedding EBV in breast milk [79], resulting in young infants being infected with EBV shortly after birth. In step 2, we ignore EBV oncogenic properties, but rather focus on the timing of infection and the dynamic relationship between malaria and EBV in infancy as a critical factor in actively contributing to the child’s survival from malaria [120], thus paradoxically increasing their long-term risk for BL from the oncogenic effects of EBV [121]. EBV carries its own viral genes that mimic human immune-regulatory genes, such as BCRF1, a homolog of human Interleukin-10. Human IL-10 is an anti-inflammatory cytokine that prevents an intense proinflammatory immune response [122], which can be lethal in malaria. Based on studies showing that recombinant BCRF1 protein inhibits the secretion of inflammatory cytokines, such as gamma interferon release, by T lymphocytes and natural killer cells, BCRF1 may modulate the host’s immune system [122]. By producing viral (v)IL-10, a protein expressed during the lytic cycle, EBV may help to modulate the child’s immune response to malaria, preventing the excessive inflammation that leads to severe complications, and helping the child survive more malaria attacks [123]. Of note, vIL-10 is expressed in BL biopsy samples [124].

Under this model, transient elevations of EBV viral load during each acute malaria attack help a child survive/recover from the short-term threat of malaria and increase their chances to grow into the high-risk age category for BL. The dynamic interaction between repeated *P. falciparum* attacks and EBV replication increases the chance of a B-cell acquiring the c-*MYC* translocation and EBV infecting that abnormal cell and delivering critical “second hit” signals to block the apoptosis triggered by negative feedback loops (Table 1). The oncogenic effects at the cellular level by EBV may be mediated by DNA methylation (e.g., via EBNA3C expression) [125] or remodeling key redox defense pathways in support of infected B-cell proliferation [126]. An important question arising from our model, potentially evaluable in pediatric malaria clinical trials, is whether the risk of severe malaria and associated mortality in children below 5 years is influenced by the timing and EBV load. A demonstration of a protective effect could give insights into the mutualistic co-evolution of *P. falciparum* and EBV to influence host and microbial fitness and biological trade-off costs.

Whether MYC translocation occurs before EBV infection, i.e., the MYC first model, or after, i.e., virus first model, is controversial [127]. Our model fits with the MYC first model, with EBV providing a second hit. Because *MYC* induces CR2 (the receptor for EBV, also called CD21), it is likely that the increased density of CR2 receptors in a *MYC*-rearranged B cell augments the probability of EBV infecting the cell [127]. Moreover, since the fate of an EBV-infected B cell depends on the type of latency established and BL is typically associated with type 1 latency, which involves expression of EBV nuclear antigen 1 (EBNA1), non-coding RNAs (EBERs), and BamH I-A rightward transcripts (BARTs) [128]. The *MYC*-first model fits with the idea that EBV infection, followed by Latency I expression, is a sufficient hit for malignancy to occur [129]. This limited expression also fits the fact that *MYC*-rearranged B cells do not progress to terminal differentiation into plasma cells and do not express the growth program (latency III), which would lead to virion production and lysis of the cell [130]. However, the first model of the virus is supported by tumor data. In the study by Grande et al., of BL genetics, they found evidence of AIDS-induced point mutations in the EBV+ BL but not in EBV- BL [113], suggesting that EBV may contribute to AIDS-induced somatic hypermutation, including rearrangements. Studies in mouse models suggest that *P. falciparum* can clearly induce AID in B-cells in the absence of EBV, but whether it can also provide survival anti-apoptotic signals triggered by over-expression of MYC in the absence of EBV is unknown. A study of DNA methylation (DNAme) profiles in individuals with hyperreactive malarial splenomegaly found no differences in the DNAme profiles in patient B cells compared to those of naive B cells [117], suggesting that *P. falciparum* does not deliver both the first and second hit. Because children who do not survive malaria die, those who recover develop a strong immune response. Although acute EBV infection is considered a risk factor for severe malaria, based on studies that mice with acute murine gammaherpesvirus 68 (MHV68) infection lose their anti-malarial humoral response [131], this hypothesis has not been evaluated in humans. Our model recasts the dynamic interaction between *P. falciparum* and EBV infections as mutualistic, conferring immediate fitness benefit against malaria mortality [132] at the cost of a small negative oncogenic risk among those who survive/recover and develop *MYC* rearranged B cells that expand and may progress to BL when they encounter secondary and tertiary hits. The severe complications of *P. falciparum*, such as cerebral malaria or severe anemia, which occur in infancy after one or two infections with highly virulent strains, impose a high population cost. We propose that EBV is a mutualistic symbiont in children lacking strain-specific anti-disease immunity [133]. Children living in high malaria transmission areas suffer a high burden of malaria from infancy, as high as 1.5 episodes per person in the first 24 weeks of life [134], underscoring this risk to life. The high risk of malaria in these early weeks of life coincides with the elevated viral load observed in infants [81], but it does not lead to a measurable increase in BL risk. By contrast, BL risk reflects heavy lifetime exposure to *P. falciparum* (risk is low below 50 lifetime infections [58]) before the risk of developing BL begins to measurably rise. Thus, while *P. falciparum*-EBV co-infection may lead to the emergence of *MYC*+ B cells. The chance of EBV infecting a cell that has acquired a c-MYC translocation imposes a constraint, which might explain the non-linear relationship with BL, i.e., absence of a high case load in the first 3 years of life, and probably requires a threshold of EBV infection of B cells for BL clones to be initiated.

Our model does not fully account for the observed decrease in BL risk after the age of 10, despite continuous and heavy exposure to malaria. We speculate that the reduction in risk may reflect the contribution of *P. falciparum* “strains” that predispose to BL, where the development of strain-specific immunity results in a corresponding decrease in BL risk. This idea is reminiscent of immunity against *P. falciparum* strains associated with severe malaria [133]. Interestingly, findings from a recent longitudinal study of malaria infection rates based in Mali may support the idea of strain-related effects [135]. Using amplicon sequencing of *P. falciparum* to quantify the molecular force of infection, the cohort participants were categorized as individuals with malaria risk (infected but without symptoms) vs. uninfected but at risk of asymptomatic infection. Using this approach, they identified a much more heterogeneous infection rate in this community cohort, with a subset of individuals apparently protected exhibiting a low molecular force of infection. It could be then that decreases in BL risk in older children are due to a decrease in the molecular force of *P. falciparum* infection as naturally acquired immunity broadens with age [136]. The decrease in EBV load observed in high transmission regions, just as the peak of BL occurs, may further accentuate the decrease in BL risk from either BL immunity or a decrease in B cell mass [94]. Alternatively, it might reflect the dynamics of B cells as discussed above.

## 10. Conclusions

The convergence of *Plasmodium falciparum* and EBV creates a perfect storm for the development of BL in SSA. This review presents an integrated model demonstrating that BL is ultimately a “tumor of survivors.” To survive the intense, potentially lethal inflammation of early childhood malaria, the host relies on EBV-mediated immune modulation (such as vIL-10 mimicry). Yet, this short-term survival mechanism paves the way for long-term oncogenesis. *P. falciparum* drives the massive B-cell expansion and AID-mediated *c-MYC* translocations, while EBV delivers the anti-apoptotic second hit that immortalizes these malignant cells. Future research must prioritize prospective cohort studies to validate these mechanistic pathways in vivo. Furthermore, the scientific community must address remaining etiological mysteries, such as the mechanisms driving male predominance in BL and the pathogenesis of adult BL in SSA. Above all, this model underscores a clear public health imperative to link programs to control the burden of *P. falciparum* with pediatric cancer prevention in regions where BL is endemic.

## Figures and Tables

**Table 1:** Joint impacts of *P. falciparum* and EBV on host immunity prior to BL onset.

Host State	*P. falciparum*	EBV	Joint Impact
**Non-immune**			
Immune modulation	Triggers intense, potentially lethal inflammation/anemia.	Modulates immune response via vIL-10, preventing severe malaria.	Improved child survival, but persistence of EBV+ B-cells.
**Pathogen dynamics**			
	Clonal, high parasitemia	Acute increases in EBV load	Uncontrolled parasitemia, strong EBV lytic replication, and immune modulation
**Strong immunity**			
Immune activation	Polyclonal B-cell activation; increased AID expression.	Establishes latent infection in an expanded B-cell pool.	Increased probability of a *c-MYC* translocation.
**Pathogen dynamics**			
	Multi-clonal, complex, low parasitemia	Stably high EBV load, in equilibrium	Chronic immune activation; EBV-*P. falciparum* equilibrium
**c-** ***MYC*** **+clones**			
Oncogenic progression	Provides secondary signals (e.g., via TLR9) that promote survival.	Delivers a “second hit” to *c-MYC* translocated cells, blocking apoptosis.	Clonal expansion and diversification with potential for malignant transformation and emergence of BL.

## Data Availability

No new data were created or analyzed in this study.

## Abbreviations

The following abbreviations are used in this manuscript:

BL	Burkitt lymphoma
AID	Activation-induced cytidine deaminase
EBV	Epstein–Barr virus
RBC	red blood cell

## References

[1] Burkitt D. (1958). A sarcoma involving the jaws in African children. Br. J. Surg..

[2] López C., Burkhardt B., Chan J.K.C., Leoncini L., Mbulaiteye S.M., Ogwang M.D., Orem J., Rochford R., Roschewski M., Siebert R. (2022). Burkitt lymphoma. Nat. Rev. Dis. Prim..

[3] Hirabayashi M., Georges D., Combes J.D., Clifford G.M. (2026). Attributable Fraction of Epstein-Barr Virus in Subtypes of Lymphoma: A Systematic Review and Global Meta-Analysis. Int. J. Cancer.

[4] Rainey J.J., Omenah D., Sumba P.O., Moormann A.M., Rochford R., Wilson M.L. (2007). Spatial clustering of endemic Burkitt’s lymphoma in high-risk regions of Kenya. Int. J. Cancer.

[5] Kafuko G.W., Burkitt D.P. (1970). Burkitt’s lymphoma and malaria. Int. J. Cancer.

[6] Haddow A.J. (1963). An Improved Map for the Study of Burkitt’s Lymphoma Syndrome in Africa. East Afr. Med. J..

[7] Robbiani D.F., Deroubaix S., Feldhahn N., Oliveira T.Y., Callen E., Wang Q., Jankovic M., Silva I.T., Rommel P.C., Bosque D. (2015). Plasmodium Infection Promotes Genomic Instability and AID-Dependent B Cell Lymphoma. Cell.

[8] Torgbor C., Awuah P., Deitsch K., Kalantari P., Duca K.A., Thorley-Lawson D.A. (2014). A multifactorial role for P. falciparum malaria in endemic Burkitt’s lymphoma pathogenesis. PLoS Pathog..

[9] Ariera B., Guyah B., Rahkola J., Arao I., Waomba K., Koech E., Samayoa-Reyes G., Sabourin K.R., Ogolla S., Rochford R. (2025). Sustained activation induced cytidine deaminase (AID) expression in B cells following Plasmodium falciparum malaria infection in Kenyan children. J. Immunol..

[10] Njie R., Bell A.I., Jia H., Croom-Carter D., Chaganti S., Hislop A.D., Whittle H., Rickinson A.B. (2009). The effects of acute malaria on Epstein-Barr virus (EBV) load and EBV-specific T cell immunity in Gambian children. J. Infect. Dis..

[11] Jayasooriya S., de Silva T.I., Njie-Jobe J., Sanyang C., Leese A.M., Bell A.I., McAulay K.A., Yanchun P., Long H.M., Dong T. (2015). Early virological and immunological events in asymptomatic epstein-barr virus infection in african children. PLoS Pathog..

[12] Lam K.M., Syed N., Whittle H., Crawford D.H. (1991). Circulating Epstein-Barr virus-carrying B cells in acute malaria. Lancet.

[13] Mbulaiteye S.M., Devesa S.S. (2022). Burkitt Lymphoma Incidence in Five Continents. Hemato.

[14] Rainey J.J., Mwanda W.O., Wairiumu P., Moormann A.M., Wilson M.L., Rochford R. (2007). Spatial distribution of Burkitt’s lymphoma in Kenya and association with malaria risk. Trop. Med. Int. Health.

[15] Morrow R.H., Pike M.C., Smith P.G., Ziegler J.L., Kisuule A. (1971). Burkitt’s lymphoma: A time-space cluster of cases in Bwamba County of Uganda. Br. Med. J..

[16] van den Bosch C.A. (2004). Is endemic Burkitt’s lymphoma an alliance between three infections and a tumour promoter?. Lancet Oncol..

[17] Mannucci S., Luzzi A., Carugi A., Gozzetti A., Lazzi S., Malagnino V., Simmonds M., Cusi M.G., Leoncini L., van den Bosch C.A. (2012). EBV Reactivation and Chromosomal Polysomies: Euphorbia tirucalli as a Possible Cofactor in Endemic Burkitt Lymphoma. Adv. Hematol..

[18] Geser A., Lenoir G.M., Anvret M., Bornkamm G., Klein G., Williams E.H., Wright D.H., De-The G. (1983). Epstein-Barr virus markers in a series of Burkitt’s lymphomas from the West Nile District, Uganda. Eur. J. Cancer Clin. Oncol..

[19] Zur Hausen H., Schulte-Holthausen H. (1970). Presence of EB virus nucleic acid homology in a “virus-free” line of Burkitt tumour cells. Nature.

[20] zur Hausen H., Schulte-Holthausen H., Klein G., Henle W., Henle G., Clifford P., Santesson L. (1970). EBV DNA in biopsies of Burkitt tumours and anaplastic carcinomas of the nasopharynx. Nature.

[21] Mpunga T., Clifford G.M., Morgan E.A., Milner D.A., de Martel C., Munyanshongore C., Muvugabigwi G., Combes J.D. (2022). Epstein-Barr virus prevalence among subtypes of malignant lymphoma in Rwanda, 2012 to 2018. Int. J. Cancer.

[22] Onwubuya I.M., Adelusola K.A., Durosinmi M.A., Sabageh D., Ezike K.N. (2015). Lymphomas in Ile-Ife, Nigeria: Immunohistochemical Characterization and Detection of Epstein-Barr virus Encoded RNA. J. Clin. Diagn. Res..

[23] Wright D.H. (1999). What is Burkitt’s lymphoma and when is it endemic?. Blood.

[24] Alaggio R., Amador C., Anagnostopoulos I., Attygalle A.D., Araujo I.B.O., Berti E., Bhagat G., Borges A.M., Boyer D., Calaminici M. (2022). The 5th edition of the World Health Organization Classification of Haematolymphoid Tumours: Lymphoid Neoplasms. Leukemia.

[25] Ogwang M.D., Bhatia K., Biggar R.J., Mbulaiteye S.M. (2008). Incidence and geographic distribution of endemic Burkitt lymphoma in northern Uganda revisited. Int. J. Cancer.

[26] Mbulaiteye S.M., Biggar R.J., Bhatia K., Linet M.S., Devesa S.S. (2009). Sporadic childhood Burkitt lymphoma incidence in the United States during 1992-2005. Pediatr. Blood Cancer.

[27] Levine P.H., Kamaraju L.S., Connelly R.R., Berard C.W., Dorfman R.F., Magrath I., Easton J.M. (1982). The American Burkitt’s Lymphoma Registry: Eight years’ experience. Cancer.

[28] Ogawa T., Kitagawa M., Hirokawa K. (2000). Age-related changes of human bone marrow: A histometric estimation of proliferative cells, apoptotic cells, T cells, B cells and macrophages. Mech. Ageing Dev..

[29] Painschab M.S., Westmoreland K.D., Kasonkanji E., Zuze T., Kaimila B., Waswa P., El-Mallawany N.K., Tomoka T., Mulenga M., Montgomery N.D. (2019). Prospective study of Burkitt lymphoma treatment in adolescents and adults in Malawi. Blood Adv..

[30] Molyneux E.M., Rochford R., Griffin B., Newton R., Jackson G., Menon G., Harrison C.J., Israels T., Bailey S. (2012). Burkitt’s lymphoma. Lancet.

[31] Atallah-Yunes S.A., Murphy D.J., Noy A. (2020). HIV-associated Burkitt lymphoma. Lancet Haematol..

[32] Korir A., Mauti N., Moats P., Gurka M.J., Mutuma G., Metheny C., Mwamba P.M., Oyiro P.O., Fisher M., Ayers L.W. (2014). Developing clinical strength-of-evidence approach to define HIV-associated malignancies for cancer registration in Kenya. PLoS ONE.

[33] Duncombe C.J., Hergott D.E.B., Staubus W., Balke-Buijs M., Kublin J.G., Duffy P.E., Healy S.A., Talley A., Jackson L., Sim B.K.L. (2026). Sex-based differences in Plasmodium infection in the control groups of controlled human malaria infection trials in malaria-naive populations in the USA and the Netherlands: A pooled analysis. Lancet Microbe.

[34] Briggs J., Teyssier N., Nankabirwa J.I., Rek J., Jagannathan P., Arinaitwe E., Bousema T., Drakeley C., Murray M., Crawford E. (2020). Sex-based differences in clearance of chronic Plasmodium falciparum infection. eLife.

[35] Snow R.W., Omumbo J.A., Lowe B., Molyneux C.S., Obiero J.O., Palmer A., Weber M.W., Pinder M., Nahlen B., Obonyo C. (1997). Relation between severe malaria morbidity in children and level of Plasmodium falciparum transmission in Africa. Lancet.

[36] Snow R.W., Marsh K. (1998). The epidemiology of clinical malaria among African children. Bull. Inst. Pasteur.

[37] Greenwood B., Marsh K., Snow R. (1991). Why do some African children develop severe malaria?. Parasitol. Today.

[38] Stevenson M.M., Riley E.M. (2004). Innate immunity to malaria. Nat. Rev. Immunol..

[39] Korbel D.S., Finney O.C., Riley E.M. (2004). Natural killer cells and innate immunity to protozoan pathogens. Int. J. Parasitol..

[40] Gupta S., Snow R.W., Donnelly C.A., Marsh K., Newbold C. (1999). Immunity to non-cerebral severe malaria is acquired after one or two infections. Nat. Med..

[41] Snow R.W., Nahlen B., Palmer A., Donnelly C.A., Gupta S., Marsh K. (1998). Risk of severe malaria among African infants: Direct evidence of clinical protection during early infancy. J. Infect. Dis..

[42] Ly A., Hansen D.S. (2019). Development of B Cell Memory in Malaria. Front. Immunol..

[43] Griffin J.T., Hollingsworth T.D., Reyburn H., Drakeley C.J., Riley E.M., Ghani A.C. (2015). Gradual acquisition of immunity to severe malaria with increasing exposure. Proc. Biol. Sci..

[44] Tran T.M., Li S., Doumbo S., Doumtabe D., Huang C.Y., Dia S., Bathily A., Sangala J., Kone Y., Traore A. (2013). An intensive longitudinal cohort study of Malian children and adults reveals no evidence of acquired immunity to Plasmodium falciparum infection. Clin. Infect. Dis..

[45] Buchwald A.G., Sorkin J.D., Sixpence A., Chimenya M., Damson M., Wilson M.L., Seydel K., Hochman S., Mathanga D., Taylor T.E. (2019). Association Between Age and Plasmodium falciparum Infection Dynamics. Am. J. Epidemiol..

[46] Maziarz M., Kinyera T., Otim I., Kagwa P., Nabalende H., Legason I.D., Ogwang M.D., Kirimunda S., Emmanuel B., Reynolds S.J. (2017). Age and geographic patterns of Plasmodium falciparum malaria infection in a representative sample of children living in Burkitt lymphoma-endemic areas of northern Uganda. Malar. J..

[47] Eldh M., Hammar U., Arnot D., Beck H.P., Garcia A., Liljander A., Mercereau-Puijalon O., Migot-Nabias F., Mueller I., Ntoumi F. (2020). Multiplicity of Asymptomatic Plasmodium falciparum Infections and Risk of Clinical Malaria: A Systematic Review and Pooled Analysis of Individual Participant Data. J. Infect. Dis..

[48] Okell L.C., Ghani A.C., Lyons E., Drakeley C.J. (2009). Submicroscopic infection in Plasmodium falciparum-endemic populations: A systematic review and meta-analysis. J. Infect. Dis..

[49] Kobayashi T., Gamboa D., Ndiaye D., Cui L., Sutton P.L., Vinetz J.M. (2015). Malaria Diagnosis Across the International Centers of Excellence for Malaria Research: Platforms, Performance, and Standardization. Am. J. Trop. Med. Hyg..

[50] Redmond L.S., Ogwang M.D., Kerchan P., Reynolds S.J., Tenge C.N., Were P.A., Kuremu R.T., Masalu N., Kawira E., Otim I. (2020). Endemic Burkitt lymphoma: A complication of asymptomatic malaria in sub-Saharan Africa based on published literature and primary data from Uganda, Tanzania, and Kenya. Malar. J..

[51] Marsh K., Snow R.W. (1997). Host-parasite interaction and morbidity in malaria endemic areas. Philos. Trans. R Soc. Lond. B Biol. Sci..

[52] Phillips R.E., Pasvol G. (1992). Anaemia of Plasmodium falciparum malaria. Baillieres Clin. Haematol..

[53] Elphinstone R.E., Riley F., Lin T., Higgins S., Dhabangi A., Musoke C., Cserti-Gazdewich C., Regan R.F., Warren H.S., Kain K.C. (2015). Dysregulation of the haem-haemopexin axis is associated with severe malaria in a case-control study of Ugandan children. Malar. J..

[54] Lindblade K.A., Steinhardt L., Samuels A., Kachur S.P., Slutsker L. (2013). The silent threat: Asymptomatic parasitemia and malaria transmission. Expert Rev. Anti Infect. Ther..

[55] Helleberg M., Goka B.Q., Akanmori B.D., Obeng-Adjei G., Rodriques O., Kurtzhals J.A. (2005). Bone marrow suppression and severe anaemia associated with persistent Plasmodium falciparum infection in African children with microscopically undetectable parasitaemia. Malar. J..

[56] Chaves A.R., Dossou Y., Djènontin A., Adimi E., Akoho R., Bailly J., Bouraïma A., Matondo D., Sissinto Y., Houinato D. (2025). Association between asymptomatic submicroscopic and microscopic malaria infections and anemia: A study in southern Benin. PLoS ONE.

[57] Chen I., Clarke S.E., Gosling R., Hamainza B., Killeen G., Magill A., O’Meara W., Price R.N., Riley E.M. (2016). Asymptomatic” Malaria: A Chronic and Debilitating Infection That Should Be Treated. PLoS Med..

[58] Broen K., Dickens J., Trangucci R., Ogwang M.D., Tenge C.N., Masalu N., Reynolds S.J., Kawira E., Kerchan P., Were P.A. (2023). Burkitt lymphoma risk shows geographic and temporal associations with Plasmodium falciparum infections in Uganda, Tanzania, and Kenya. Proc. Natl. Acad. Sci. USA.

[59] Scholzen A., Sauerwein R.W. (2013). How malaria modulates memory: Activation and dysregulation of B cells in Plasmodium infection. Trends Parasitol..

[60] Parroche P., Lauw F.N., Goutagny N., Latz E., Monks B.G., Visintin A., Halmen K.A., Lamphier M., Olivier M., Bartholomeu D.C. (2007). Malaria hemozoin is immunologically inert but radically enhances innate responses by presenting malaria DNA to Toll-like receptor 9. Proc. Natl. Acad. Sci. USA.

[61] Bai L., Chen W., Chen J., Li W., Zhou L., Niu C., Han W., Cui J. (2017). Heterogeneity of Toll-like receptor 9 signaling in B cell malignancies and its potential therapeutic application. J. Transl. Med..

[62] Peng S.L. (2005). Signaling in B cells via Toll-like receptors. Curr. Opin. Immunol..

[63] He B., Qiao X., Cerutti A. (2004). CpG DNA induces IgG class switch DNA recombination by activating human B cells through an innate pathway that requires TLR9 and cooperates with IL-10. J. Immunol..

[64] Dalldorf G., Linsell C.A., Barnhart F.E., Martyn R. (1964). An Epidemiologic Approach to the Lymphomas of African Children and Burkitt’s Sacroma of the Jaws. Perspect. Biol. Med..

[65] Ziegler J.L., Bluming A.Z., Morrow R.H., Cohen M.H., Fife E.H., Finerty J.F., Woods R. (1972). Burkitt’s lymphoma and malaria. Trans. R. Soc. Trop. Med. Hyg..

[66] Morrow R.H. (1985). Epidemiological evidence for the role of falciparum malaria in the pathogenesis of Burkitt’s lymphoma. IARC Sci. Publ..

[67] Carpenter L.M., Newton R., Casabonne D., Ziegler J., Mbulaiteye S., Mbidde E., Wabinga H., Jaffe H., Beral V. (2008). Antibodies against malaria and Epstein-Barr virus in childhood Burkitt lymphoma: A case-control study in Uganda. Int. J. Cancer.

[68] Mutalima N., Molyneux E., Jaffe H., Kamiza S., Borgstein E., Mkandawire N., Liomba G., Batumba M., Lagos D., Gratrix F. (2008). Associations between Burkitt lymphoma among children in Malawi and infection with HIV, EBV and malaria: Results from a case-control study. PLoS ONE.

[69] Aka P., Vila M.C., Jariwala A., Nkrumah F., Emmanuel B., Yagi M., Palacpac N.M., Periago M.V., Neequaye J., Kiruthu C. (2013). Endemic Burkitt lymphoma is associated with strength and diversity of Plasmodium falciparum malaria stage-specific antigen antibody response. Blood.

[70] Derkach A., Otim I., Pfeiffer R.M., Onabajo O.O., Legason I.D., Nabalende H., Ogwang M.D., Kerchan P., Talisuna A.O., Ayers L.W. (2019). Associations between IgG reactivity to Plasmodium falciparum erythrocyte membrane protein 1 (PfEMP1) antigens and Burkitt lymphoma in Ghana and Uganda case-control studies. EBioMedicine.

[71] Perez-Mazliah D., Langhorne J. (2014). CD4 T-cell subsets in malaria: TH1/TH2 revisited. Front Immunol..

[72] Bedsaul-Fryer J.R., Mbulaiteye S.M. (2026). Selective Immune Tolerance: A Unifying Mechanism Linking Heavy Malaria Exposure, Waning Vaccine Antibodies, and Burkitt Lymphoma. J. Infect. Dis..

[73] Geser A., Brubaker G., Draper C.C. (1989). Effect of a malaria suppression program on the incidence of African Burkitt’s lymphoma. Am. J. Epidemiol..

[74] World Health Organization (2020). World Malaria Report 2020: 20 Years of Global Progress and Challenges.

[75] Weiss D.J., Dzianach P.A., Saddler A., Lubinda J., Browne A., McPhail M., Rumisha S.F., Sanna F., Gelaw Y., Kiss J.B. (2025). Mapping the global prevalence, incidence, and mortality of Plasmodium falciparum and Plasmodium vivax malaria, 2000-22: A spatial and temporal modelling study. Lancet.

[76] Mwaniki M.K., Mohammed S., Kariuki N., Wamalwa D.C., Mutisya F., Newton C.R. (2025). A “Familiar Foe Revisited”: Examining the relationship between endemic Burkitt’s lymphoma and changing malaria admissions in the coastal region of Kenya. BMC Cancer.

[77] Schmit N., Kaur J., Aglago E.K. (2024). Mosquito Bed Net Use and Burkitt Lymphoma Incidence in Sub-Saharan Africa: A Systematic Review and Meta-Analysis. JAMA Netw. Open.

[78] Niederman J.C., Miller G., Pearson H.A., Pagano J.S., Dowaliby J.M. (1976). Infectious mononucleosis. Epstein-Barr-virus shedding in saliva and the oropharynx. N. Engl. J. Med..

[79] Daud I.I., Coleman C.B., Smith N.A., Ogolla S., Simbiri K., Bukusi E.A., Ng’ang’a Z.W., Sumba P.O., Vulule J., Ploutz-Snyder R. (2015). Breast Milk as a Potential Source of Epstein-Barr Virus Transmission Among Infants Living in a Malaria-Endemic Region of Kenya. J. Infect. Dis..

[80] Dunmire S.K., Hogquist K.A., Balfour H.H. (2015). Infectious Mononucleosis. Curr. Top. Microbiol. Immunol..

[81] Piriou E., Asito A.S., Sumba P.O., Fiore N., Middeldorp J.M., Moormann A.M., Ploutz-Snyder R., Rochford R. (2012). Early age at time of primary Epstein-Barr virus infection results in poorly controlled viral infection in infants from Western Kenya: Clues to the etiology of endemic Burkitt lymphoma. J. Infect. Dis..

[82] Biggar R.J., Henle W., Fleisher G., Böcker J., Lennette E.T., Henle G. (1978). Primary Epstein-Barr virus infections in African infants. I. Decline of maternal antibodies and time of infection. Int. J. Cancer.

[83] Reynaldi A., Schlub T.E., Chelimo K., Sumba P.O., Piriou E., Ogolla S., Moormann A.M., Rochford R., Davenport M.P. (2016). Impact of Plasmodium falciparum Coinfection on Longitudinal Epstein-Barr Virus Kinetics in Kenyan Children. J. Infect. Dis..

[84] Piriou E., Kimmel R., Chelimo K., Middeldorp J.M., Odada P.S., Ploutz-Snyder R., Moormann A.M., Rochford R. (2009). Serological evidence for long-term Epstein-Barr virus reactivation in children living in a holoendemic malaria region of Kenya. J. Med. Virol..

[85] Donati D., Espmark E., Kironde F., Mbidde E.K., Kamya M., Lundkvist A., Wahlgren M., Bejarano M.T., Falk K.I. (2006). Clearance of circulating Epstein-Barr virus DNA in children with acute malaria after antimalaria treatment. J. Infect. Dis..

[86] Rasti N., Falk K.I., Donati D., Gyan B.A., Goka B.Q., Troye-Blomberg M., Akanmori B.D., Kurtzhals J.A., Dodoo D., Consolini R. (2005). Circulating epstein-barr virus in children living in malaria-endemic areas. Scand. J. Immunol..

[87] Samayoa-Reyes G., Weigel C., Koech E., Waomba K., Jackson C., Onditi I.A., Sabourin K.R., Kenney S., Baiocchi R.A., Oakes C.C. (2024). Effect of Malaria Infection on Epstein-Barr Virus Persistence in Kenyan Children. J. Infect. Dis..

[88] Yone C.L., Kube D., Kremsner P.G., Luty A.J. (2006). Persistent Epstein-Barr viral reactivation in young African children with a history of severe Plasmodium falciparum malaria. Trans. R Soc. Trop. Med. Hyg..

[89] Jayasooriya S., Hislop A., Peng Y., Croom-Carter D., Jankey Y., Bell A., Dong T., Rowland-Jones S., Rickinson A., Walther M. (2012). Revisiting the effect of acute P. falciparum malaria on Epstein-Barr virus: Host balance in the setting of reduced malaria endemicity. PLoS ONE.

[90] Babcock G.J., Decker L.L., Freeman R.B., Thorley-Lawson D.A. (1999). Epstein-barr virus-infected resting memory B cells, not proliferating lymphoblasts, accumulate in the peripheral blood of immunosuppressed patients. J. Exp. Med..

[91] Thorley-Lawson D.A. (2015). EBV Persistence--Introducing the Virus. Curr. Top. Microbiol. Immunol..

[92] Nash S.D., Prevots D.R., Kabyemela E., Khasa Y.P., Lee K.L., Fried M., Duffy P.E. (2017). A Malaria-Resistant Phenotype with Immunological Correlates in a Tanzanian Birth Cohort Exposed to Intense Malaria Transmission. Am. J. Trop. Med. Hyg..

[93] Mwanda O.W., Rochford R., Moormann A.M., Macneil A., Whalen C., Wilson M.L. (2004). Burkitt’s lymphoma in Kenya: Geographical, age, gender and ethnic distribution. East Afr. Med. J..

[94] Moormann A.M., Chelimo K., Sumba O.P., Lutzke M.L., Ploutz-Snyder R., Newton D., Kazura J., Rochford R. (2005). Exposure to holoendemic malaria results in elevated Epstein-Barr virus loads in children. J. Infect. Dis..

[95] Mooney J.P., Barry A., Gonçalves B.P., Tiono A.B., Awandu S.S., Grignard L., Drakeley C.J., Bottomley C., Bousema T., Riley E.M. (2018). Haemolysis and haem oxygenase-1 induction during persistent “asymptomatic” malaria infection in Burkinabé children. Malar. J..

[96] Watanabe-Matsui M., Muto A., Matsui T., Itoh-Nakadai A., Nakajima O., Murayama K., Yamamoto M., Ikeda-Saito M., Igarashi K. (2011). Heme regulates B-cell differentiation, antibody class switch, and heme oxygenase-1 expression in B cells as a ligand of Bach2. Blood.

[97] Igarashi K., Ochiai K., Itoh-Nakadai A., Muto A. (2014). Orchestration of plasma cell differentiation by Bach2 and its gene regulatory network. Immunol. Rev..

[98] Laichalk L.L., Thorley-Lawson D.A. (2005). Terminal differentiation into plasma cells initiates the replicative cycle of Epstein-Barr virus in vivo. J. Virol..

[99] Burnet A.M., Brunetti T., Rochford R. (2023). Hemin treatment drives viral reactivation and plasma cell differentiation of EBV latently infected B cells. PLoS Pathog..

[100] Chene A., Donati D., Guerreiro-Cacais A.O., Levitsky V., Chen Q., Falk K.I., Orem J., Kironde F., Wahlgren M., Bejarano M.T. (2007). A molecular link between malaria and Epstein-Barr virus reactivation. PLoS Pathog..

[101] Chene A., Donati D., Orem J., Mbidde E.R., Kironde F., Wahlgren M., Bejarano M.T. (2009). Endemic Burkitt’s lymphoma as a polymicrobial disease: New insights on the interaction between Plasmodium falciparum and Epstein-Barr virus. Semin. Cancer Biol..

[102] Moormann A.M., Bailey J.A., Rochford R. (2025). Burkitt Lymphoma. Curr. Top. Microbiol. Immunol..

[103] Ariera B.O., Guyah B., Onditi I., Waomba K., Koech E., Sabourin K.R., Samayoa-Reyes G., Rochford R., Ogolla S. (2025). CD4 and CD8 T-cell response is dominated by IL-10-secreting cells in children with uncomplicated Plasmodium falciparum malaria. Immunohorizons.

[104] Feng Y., Seija N., Di Noia J.M., Martin A. (2020). AID in Antibody Diversification: There and Back Again. Trends Immunol..

[105] Robbiani D.F., Bothmer A., Callen E., Reina-San-Martin B., Dorsett Y., Difilippantonio S., Bolland D.J., Chen H.T., Corcoran A.E., Nussenzweig A. (2008). AID is required for the chromosomal breaks in c-myc that lead to c-myc/IgH translocations. Cell.

[106] Wilmore J.R., Maue A.C., Rochford R. (2016). Plasmodium chabaudi infection induces AID expression in transitional and marginal zone B cells. Immun. Inflamm. Dis..

[107] Vecchio E., Fiume G., Correnti S., Romano S., Iaccino E., Mimmi S., Maisano D., Nisticò N., Quinto I. (2020). Insights about MYC and Apoptosis in B-Lymphomagenesis: An Update from Murine Models. Int. J. Mol. Sci..

[108] Nduati E., Gwela A., Karanja H., Mugyenyi C., Langhorne J., Marsh K., Urban B.C. (2011). The plasma concentration of the B cell activating factor is increased in children with acute malaria. J. Infect. Dis..

[109] Scholzen A., Teirlinck A.C., Bijker E.M., Roestenberg M., Hermsen C.C., Hoffman S.L., Sauerwein R.W. (2014). BAFF and BAFF receptor levels correlate with B cell subset activation and redistribution in controlled human malaria infection. J. Immunol..

[110] Mackay F., Tangye S.G. (2004). The role of the BAFF/APRIL system in B cell homeostasis and lymphoid cancers. Curr. Opin. Pharmacol..

[111] Sakai J., Akkoyunlu M. (2017). The Role of BAFF System Molecules in Host Response to Pathogens. Clin. Microbiol. Rev..

[112] Paschos K., Smith P., Anderton E., Middeldorp J.M., White R.E., Allday M.J. (2009). Epstein-barr virus latency in B cells leads to epigenetic repression and CpG methylation of the tumour suppressor gene Bim. PLoS Pathog..

[113] Grande B.M., Gerhard D.S., Jiang A., Griner N.B., Abramson J.S., Alexander T.B., Allen H., Ayers L.W., Bethony J.M., Bhatia K. (2019). Genome-wide discovery of somatic coding and non-coding mutations in pediatric endemic and sporadic Burkitt lymphoma. Blood.

[114] He B., Raab-Traub N., Casali P., Cerutti A. (2003). EBV-encoded latent membrane protein 1 cooperates with BAFF/BLyS and APRIL to induce T cell-independent Ig heavy chain class switching. J. Immunol..

[115] Kalchschmidt J.S., Bashford-Rogers R., Paschos K., Gillman A.C., Styles C.T., Kellam P., Allday M.J. (2016). Epstein-Barr virus nuclear protein EBNA3C directly induces expression of AID and somatic mutations in B cells. J. Exp. Med..

[116] Wilmore J.R., Asito A.S., Wei C., Piriou E., Sumba P.O., Sanz I., Rochford R. (2015). AID expression in peripheral blood of children living in a malaria holoendemic region is associated with changes in B cell subsets and Epstein-Barr virus. Int. J. Cancer.

[117] Glaser S., Wagener R., Kretzmer H., Lopez C., Baptista M.J., Bens S., Bernhart S., Bhatia K., Borkhardt A., Elgaafary S. (2025). Subtyping Burkitt Lymphoma by DNA Methylation. Genes Chromosom. Cancer.

[118] Nagy N., Klein G., Klein E. (2009). To the genesis of Burkitt lymphoma: Regulation of apoptosis by EBNA-1 and SAP may determine the fate of Ig-myc translocation carrying B lymphocytes. Semin. Cancer Biol..

[119] Venkatesan P. (2024). The 2023 WHO World malaria report. Lancet Microbe.

[120] Moore K.W., Vieira P., Fiorentino D.F., Trounstine M.L., Khan T.A., Mosmann T.R. (1990). Homology of cytokine synthesis inhibitory factor (IL-10) to the Epstein-Barr virus gene BCRFI. Science.

[121] Watier H., Auriault C., Capron A. (1993). Does Epstein-Barr virus infection confer selective advantage to malaria-infected children?. Lancet.

[122] Hsu D.-H., Malefyt R.d.W., Fiorentino D.F., Dang M.-N., Vieira P., deVries J., Spits H., Mosmann T.R., Moore K.W. (1990). Expression of Interleukin-10 Activity by Epstein-Barr Virus Protein BCRF1. Science.

[123] Kurtzhals J.A., Adabayeri V., Goka B.Q., Akanmori B.D., Oliver-Commey J.O., Nkrumah F.K., Behr C., Hviid L. (1998). Low plasma concentrations of interleukin 10 in severe malarial anaemia compared with cerebral and uncomplicated malaria. Lancet.

[124] Xue S.A., Labrecque L.G., Lu Q.L., Ong S.K., Lampert I.A., Kazembe P., Molyneux E., Broadhead R.L., Borgstein E., Griffin B.E. (2002). Promiscuous expression of Epstein-Barr virus genes in Burkitt’s lymphoma from the central African country Malawi. Int. J. Cancer.

[125] Zhang L., Wang R., Xie Z. (2022). The roles of DNA methylation on the promotor of the Epstein-Barr virus (EBV) gene and the genome in patients with EBV-associated diseases. Appl. Microbiol. Biotechnol..

[126] Burton E.M., Mitra B., Guo R., Asara J.M., Gewurz B.E. (2026). Epstein-Barr Virus Latent Membrane Protein 1 Suppresses Ferroptosis via Pentose Phosphate Pathway and Glutathione Metabolism. bioRxiv.

[127] Molina E., García-Gutiérrez L., Junco V., Perez-Olivares M., de Yébenes V.G., Blanco R., Quevedo L., Acosta J.C., Marín A.V., Ulgiati D. (2023). MYC directly transactivates CR2/CD21, the receptor of the Epstein–Barr virus, enhancing the viral infection of Burkitt lymphoma cells. Oncogene.

[128] Navari M., Etebari M., De Falco G., Ambrosio M.R., Gibellini D., Leoncini L., Piccaluga P.P. (2015). The presence of Epstein-Barr virus significantly impacts the transcriptional profile in immunodeficiency-associated Burkitt lymphoma. Front. Microbiol..

[129] Humme S., Reisbach G., Feederle R., Delecluse H.J., Bousset K., Hammerschmidt W., Schepers A. (2003). The EBV nuclear antigen 1 (EBNA1) enhances B cell immortalization several thousandfold. Proc. Natl. Acad. Sci. USA.

[130] Thorley-Lawson D.A., Gross A. (2004). Persistence of the Epstein-Barr virus and the origins of associated lymphomas. N. Engl. J. Med..

[131] Matar C.G., Anthony N.R., O’Flaherty B.M., Jacobs N.T., Priyamvada L., Engwerda C.R., Speck S.H., Lamb T.J. (2015). Gammaherpesvirus Co-infection with Malaria Suppresses Anti-parasitic Humoral Immunity. PLoS Pathog..

[132] Roossinck M.J. (2011). The good viruses: Viral mutualistic symbioses. Nat. Rev. Microbiol..

[133] Gupta S., Hill A.V., Kwiatkowski D., Greenwood A.M., Greenwood B.M., Day K.P. (1994). Parasite virulence and disease patterns in Plasmodium falciparum malaria. Proc. Natl. Acad. Sci. USA.

[134] Aguti M., Nankabirwa J.I., Kizza J., Kakuru A., Ssemukuye T., Adrama H., Olwoch P., Opira B., Odongo B., Camanag K. (2026). Natural History and the Burden of Malaria During the First Year of Life in the High-Transmission Setting of Uganda. Am. J. Trop. Med. Hyg..

[135] LaVerriere E., Johnson Z.M., Shieh M., Nziza N., Alter G., Buckee C.O., Crompton P.D., Traore B., Tran T.M., Neafsey D.E. (2025). Marked heterogeneity in malaria infection rate in a Malian longitudinal cohort. Nat. Commun..

[136] Cham G.K., Turner L., Kurtis J.D., Mutabingwa T., Fried M., Jensen A.T., Lavstsen T., Hviid L., Duffy P.E., Theander T.G. (2010). Hierarchical, domain type-specific acquisition of antibodies to Plasmodium falciparum erythrocyte membrane protein 1 in Tanzanian children. Infect. Immun..

